# Hernia

**Published:** 1829-04

**Authors:** 


### HERNIA.

*Strangulated Umbilical Hernia. Cessation of Vomiting for eight Days. Operation performed fifteen Days after the occurrence of the first Symptoms of Strangulation. Fatal Result.* (Hôtel Dieu.)

A woman, sixty-two years of age, the mother of six children, had had for fifteen years an umbilical hernia, about the size of a goose's egg. She had always worn a bandage, which had not, however, been properly applied. The rupture had never been entirely reduced. The patient had been subject to attacks of colic, but never to vomiting. She was admitted January 29th, 1829. Fifteen days before she had suffered from colic, shiverings, hiccup, nausea, and vomiting, at first of mucous and alimentary, and afterwards of bilious and fecal matter. The symptoms ceased on the 24th, but she had no evacuation from the bowels.

The day after her admission into the hospital, the tumor was tense, painful to the touch, of considerable size, being four inches in diameter. It surrounded the navel. The pulse was strong, face flushed. The patient replied to any questions that were put to her with hesitation, but still in a rational manner. No colic nor vomiting; no evacuation from the bowels. M. Dupuytren did not think himself called upon to operate, because there was no other symptom of strangulation than the constipated state of the bowels.* He ordered fifteen leeches to be applied to the tumor, and afterwards emollient cataplasms; warm bath; a purgative clyster; veal broth.

31st.—Patient extremely weak. Tumor in the same state. Pulse quick and feeble; countenance much changed. Has not vomited. Has discharged flatus freely from the bowels, but no feces.—V.S. from the arm.

In the afternoon, she was attacked with hiccup, nausea, and vomiting of stercoraceous matter, and colic. Erysipelatous inflammation of the hypogastric region.

M. Sanson operated three hours afterwards. He made a long longitudinal incision, and laid bare the sac, which was much thickened, particularly at its lower part. It contained a portion of intestine about four inches long, which appeared to be a part of

* Were the tension and painful condition of the tumor not symptoms of strangulation? We are almost inclined to believe that the opinion of M. Dupuytren must have been mistaken, and consequently misstated.—Editors.

the transverse arch of the colon. There were numerous adhesions between the parts, and it was thought there was a portion of omentum at the bottom of the wound. The strangulated parts were relieved by an incision upwards and towards the left side. The intestine, which was of a deep red colour, was reduced. In three-quarters of an hour the patient again vomited fecal matter. Clysters were administered, and five or six evacuations from the bowels followed.

February 1st.—Twenty leeches were applied to the hypogastric region, over the part affected with erysipelas. Countenance of the patient much altered; pulse thready. She could scarcely articulate. Died in the night.

*Dissection, thirty hours after death.*—Abdomen: The convolutions of the intestines were of a deep red colour, and were surrounded by purulent matter; flakes of false membrane were observed upon their surface. The parts constituting the hernia had escaped from the abdominal cavity through a separation of the linea alba, above the umbilicus. The portion of intestine which had been reduced was a part of the arch of the colon. A part of the omentum was still in the opening, and adhered strongly to the edges of it.

*Observations*, (by the relater of the case, in the *Journal Hebdomadaire*.)—This case is very curious, inasmuch as the principal symptoms of strangulation did not reappear for eight days after they had ceased at first. The woman vomited fecal matter from the 16th to the 24th of January: vomiting then ceased, and did not return till eight days after. M. Dupuytren remarked that it was the first case of the kind he had observed. He had known the vomiting cease for two or three days, but never for so long a period as in this instance, while a portion of the intestine was still strangulated.

This case appears to us "très curieuse," for other reasons than those assigned by the relater of it. Surely the symptoms did demand, and urgently too, the immediate performance of the operation the day after the patient was admitted into the hospital. M. Dupuytren, it seems, entertained a contrary opinion; but the reasons for which he is said to have decided upon deferring the operation seem to us so decidedly erroneous, and so completely contradicted by the condition of the patient at the moment, that we must suspect the report of the case upon this point to be inaccurate.—Editors.

